# *CGN* Correlates With the Prognosis and Tumor Immune Microenvironment in Clear Cell Renal Cell Carcinoma

**DOI:** 10.3389/fmolb.2022.758974

**Published:** 2022-02-09

**Authors:** Zijian Tian, Lingfeng Meng, Xin Wang, Tongxiang Diao, Maolin Hu, Miao Wang, Yaqun Zhang, Ming Liu, Jianye Wang

**Affiliations:** ^1^ Department of Urology, Beijing Hospital, National Center of Gerontology, Institute of Geriatric Medicine, Chinese Academy of Medical Sciences, Beijing, China; ^2^ Graduate School of Peking Union Medical College, Chinese Academy of Medical Sciences, Beijing, China

**Keywords:** CGN, clear cell renal carcinoma, survival analysis, prognosis, tumor immune microenvironment

## Abstract

This study aimed to screen and verify the important prognostic genes related to clear cell renal cell carcinoma (ccRCC) and further analyze their relationship with the immune microenvironment. Gene expression profiles from the TCGA-KIRC, GSE46699, GSE36895, and GSE16449 datasets were utilized to explore differentially co-expressed genes in ccRCC. We screened 124 differentially co-expressed genes using a weighted gene co-expression network and differential gene expression analyses. Univariate and multivariate Cox survival analyses revealed that the expressions of genes *CGN, FECH, UCHL1*, and *WT1* were independently related to the overall survival of ccRCC patients. Kaplan–Meier survival analysis was performed, and *CGN* was found to have the strongest correlation with the prognosis of ccRCC patients and was consequently selected for further analyses and experimental verification. The results showed that NK cell activation, resting dendritic cells, resting monocytes, and resting mast cells were positively correlated with *CGN* expression; CD4^+^ memory activated T cells, regulatory T cells, and M0 macrophages were negatively correlated with *CGN* expression. Finally, using western blotting and reverse transcription polymerase chain reaction, we verified that the *CGN* protein level was down-regulated in ccRCC samples, which was consistent with the mRNA levels. *CGN* was thus identified as diagnosis and prognosis biomarker for ccRCC and is related to the immune microenvironment.

## Introduction

Renal cell carcinoma (RCC) is one of the 10 most common cancers; 73,750 new cases of RCC and 14,830 RCC deaths were reported in 2020 in the United States (Siegel et al., 2020). Almost one-third of RCC patients have metastatic spread at the onset of disease, and nearly half of these patients die from the disease (Bhatt and Finelli, 2014; Bray et al., 2018). The histological subtypes of RCC are highly heterogeneous in terms of their biological characteristics and treatment results. The most common (70–80%) subtype is clear cell renal cell carcinoma (ccRCC), which is also the most aggressive (Linehan, 2012). The clinical manifestations of ccRCC are subtle. A triad with typical low back pain, visible hematuria, and a palpable abdominal mass is rare (6–10%) and is mostly associated with aggressive histology and advanced disease (Lee et al., 2002; Patard et al., 2003). Therefore, development of strategies for the early identification of ccRCC has been the focus of research in recent years.

Weighted gene co-expression network analysis (WGCNA) is a systems biology method that delineates gene association patterns between different samples and can be used to identify highly coordinated gene sets. This method considers not only the co-expression pattern between two genes but also the overlap of adjacent genes and identifies candidate biomarker genes or therapeutic targets based on the interconnectivity of gene sets and the association between gene sets and the phenotype (Langfelder and Horvath, 2008; Yang et al., 2014; Tian et al., 2021). Differential gene expression analysis primarily uses statistical methods to identify the differential genes related to the conditions between two groups and further analyzes the biological significance of the identified differential genes (San Segundo-Val and Sanz-Lozano, 2016).

Although several genes have been associated with the occurrence and development of renal cancer, such as *BAP1, BIRC5, CXCR4,* and *SETD2* (Petitprez et al., 2021), there are other undiscovered genes that can be used as markers of ccRC*C,* which can provide new insights into the mechanism of occurrence and development of ccRCC. This study was intended at combining the results of WGCNA and differential gene expression analysis and then verifying the results experimentally to improve the recognition ability of highly related genes as candidate biomarkers. By analyzing the differentially co-expressed genes in ccRCC, this study provides a new idea for exploring the pathogenesis of ccRCC.

## Methods

### Data and Sample Collection

RNA sequencing data of 72 normal renal samples and 539 ccRCC samples with corresponding somatic mutation data and clinical data were obtained from The Cancer Genome Atlas (TCGA) database (https://cancerg enome.nih.gov/). Microarray data sets (GSE46699 (63 normal renal and 67 ccRCC samples), GSE36895 (23 normal renal and 29 ccRCC samples), and GSE16449 (18 normal renal and 52 ccRCC samples)) and clinical data (GSE3, GSE22541 and GSE29609) were downloaded from the Gene Expression Omnibus (GEO) database (http://www.ncbi.nlm.nih.gov/geo/).

Clinical and pathological data of 59 patients undergoing renal cancer resection in Beijing Hospital were collected retrospectively and further screening and analysis were performed. The inclusion criteria were as follows: (Siegel et al., 2020): patients who underwent nephrectomy and pathological diagnosis of ccRCC in Beijing Hospital; (Bray et al., 2018); surgery occurred between October 2019 and June 2021; and (Bhatt and Finelli, 2014) patients were ≥18 years old. The exclusion criterion was: (Siegel et al., 2020) no fresh tissue samples available. All fresh tissue samples were collected immediately after surgical resection, quickly frozen in liquid nitrogen and stored at −80 °C until RNA was extracted from the samples. Reverse transcription polymerase chain reaction (RT-PCR) and western blotting (WB) were performed on the fresh ccRCC samples to assess *CGN* expression. All protocols were approved by the Research Ethics Committee of Beijing Hospital.

### Weighted Co-expression Network Construction and Hub Module Screening

To clarify the association between genes, a weighted gene co-expression network was constructed for the hierarchical clustering of all genes subjected to co-expression analysis. The protocol used was as follows: (Siegel et al., 2020): The Pearson correlation coefficient between genes was determined. (Bray et al., 2018). A weighted adjacency matrix *α*_mn_ = |c_mn_|^β^ was constructed, where *α*_mn_ represents the adjacency matrix between gene m and gene n; c_mn_ represents the Pearson correlation coefficient between gene m and gene n; and *β* is the soft power value. The pickSoftThreshold function of the WGCNA software package was used to select the appropriate soft threshold power *β*. An appropriate soft power value *β* can ensure that the network is in accordance with a standard non-scale network to achieve a scale-free topology. (Bhatt and Finelli, 2014). The adjacency matrix was converted into a topological overlap matrix (TOM), and the dissimilarity matrix between genes dissTOM = 1-TOM was calculated. (Linehan, 2012). Hierarchical clustering was performed using dissTOM such that genes with similar expression patterns are placed in the same gene module. (Lee et al., 2002). The minimum number of module genes was set at 50, and the dynamic hybrid cutting algorithm was used to obtain the gene modules and merge modules that were highly similar. (Patard et al., 2003). The Pearson correlation coefficient between each module and the disease traits was determined, and the *p* value was used to determine the hub module. The genes in the gene module with the highest association coefficient were used as candidate prognostic molecular markers and were included in subsequent analyses.

### Screening of Differentially Expressed Genes and Intersection Genes

The DEGs were screened from the normal and tumor tissue groups. The screening criteria were false discovery rate (FDR) < 0.05 and │log_2_ FC│≥1.0, where FC is the fold change that is the multiple of the differential expression levels between the two groups. Subsequently, the genes of the hub module and the DEGs in the weighted co-expression network were intersected to identify the differentially co-expressed genes, which were visualized using the R package VennDiagram. A hypergeometric test was used to test the statistical significance of Venn diagram, and *p* < 0.05 was considered statistically significant.

### GO and Pathway Analysis

The clusterProfiler package in the R software was used to perform a GO enrichment analysis on the intersecting genes. A recently developed algorithm based on the pathway topology to identify the functional roles of pathway components was used for pathway annotations (Sorokin et al., 2021).

### Survival Analysis

R language survival package was used to perform a survival analysis on the intersecting genes. From the TCGA database, univariate Cox survival analysis-screened genes with a FDR<0.05 were included in the multivariate Cox survival analysis to obtain independent prognostic genes. A Kaplan–Meier (KM) survival analysis was then performed to screen for the most relevant genes for prognosis. Finally, TCGA, GSE3, GSE22541 and GSE29609 datasets were integrated with the application of the “ComBat” algorithm to eliminate batch effect, and the prognostic effect of *CGN* was verified.

### Association Between *CGN* and Clinical Traits

To explore the relationship between *CGN* and clinical traits, *CGN* expression level was divided into two groups according to the median value. The Wilcox test was used to explore the relationship between *CGN* and age, sex, T stage, N stage, M stage, tumor grade, and tumor stage. Statistical significance was set at *p* < 0.05.

### Association between *CGN* expression and tumor-infiltrating immune cells

The “CIBERSORT” R package was used to analyze the infiltrating immune components of each sample, and the Pearson correlation analysis was used to determine the linear relationship between the 22 immune cell types (naïve B cells, memory B cells, plasma cells, CD8 T cells, CD4 naïve T cells, CD4 memory resting T cells, CD4 memory activated T cells, follicular helper T cells, regulatory T cells, gamma delta T cells, resting NK cells, activated NK cells, Monocytes, M0 macrophages, M1 macrophages, M2 macrophages, resting dendritic cells, activated dendritic cells, resting mast cells, activated mast cells, eosinophils, and neutrophils) and *CGN*. We further divided the expression of *CGN* into high and low expression groups according to the median value, and the Wilcoxon test was used to detect the immune cells related to *CGN*. Finally, the intersection of the two results identified the immune cells that are most closely related to *CGN*.

### Association analysis between *CGN* expression and tumor mutation burden and PD-L1 expression

The linear relationship between *CGN* and TMB in the TCGA dataset was obtained by the Pearson correlation analysis. Then, *CGN* was divided into high and low expression groups based on the median value. The Wilcoxon test was used to compare the relationship between these two groups and TMB. TMB was divided into two groups based on the median value and a KM survival analysis was performed. The KM method and log-rank test shows the survival curves of *CGN* stratification and TMB stratification. The Wilcoxon test was used to compare the relationship between high and low expressions *CGN* and PD-L1 of TCGA and GSE16449.

### Association Analysis Between *CGN* Expression and Existing ccRCC Biomarkers

The linear relationship between *CGN* and existing ccRCC biomarkers (*BAP1, BIRC5, CXCR4,* and *SETD2*) in the TCGA dataset was obtained by the Pearson correlation analysis. Statistical significance was set at *p* < 0.05.

### Human Protein Atlas Database and Experimental Validation

Protein expression of the *CGN* gene in the tumor and normal tissues was obtained using the HPA database (https://www.proteinatlas.org/).

The mRNA transcription level of *CGN* in the tumor and normal tissues of the ccRCC patients that were preserved in our hospital was analyzed. TRI Reagent (Sigma) was used to extract the total RNA from the specimens. A NanoDrop^®^ ND-1000 spectrophotometer was used to determine the RNA concentration and purity. Total RNA was reverse-transcribed into cDNA using SuperScriptTM III Reverse transcriptase (Invitrogen), and the cDNA was used as a template to detect the expression of each gene by RT-PCR. The primer sequences used were as follows:

*CGN* forward: 5′-CAG​GGC​ATT​GGC​AGA​GTA​TGT-3′;

*CGN* reverse: 5′-CCT​CAA​CCT​GGC​GAG​TAT​CT-3′; *β*-actin forward: 5′-GTG​GCC​GAG​GAC​TTT​GAT​TG-3′;

β-actin reverse: 5′-CCT​GTA​ACA​ACG​CAT​CTC​ATA​TT-3′.

Total protein was extracted with pre-chilled RIPA lysis buffer and was quantified using a BCA protein assay kit (Cwbiotech), according to the manufacturer’s guidelines. SDS-PAGE was performed on the protein samples and blocked at room temperature after transfer to PVDF (Millipore) membranes with added anti-CGN (Sigma) primary antibody diluted 1:1000 and incubated overnight. A secondary antibody (goat anti-rabbit IgG (H + L), HRP 1:10,000, Jackson) was added, and the samples were incubated for 1 h, washed with TBST washing solution, reacted with ECL reagent (Millipore), and visualized. The software used for grayscale analysis of the image was Gel Image System ver.4.00 (Tanon, China).

### Statistical Analysis

Wilcox test is a non-parametric statistical hypothesis test mainly used for comparison between two groups. Pearson correlation analysis is used to compare the association between two parameters. Univariate and multivariate Cox regression analysis were used to study the relationship between gene expression and the overall survival (OS) rate of patients. The expression level of *CGN* was divided into a high-risk group and a low-risk group according to the median, and the OS of the patients was analyzed by the Kaplan-Meier method. *p* < 0.05 was considered statistically significant, and all statistical analyses were performed using the R software (version 4.1.0).

## Results

### Construction of a Weighted Gene Co-expression Module

**FIGURE 1:**
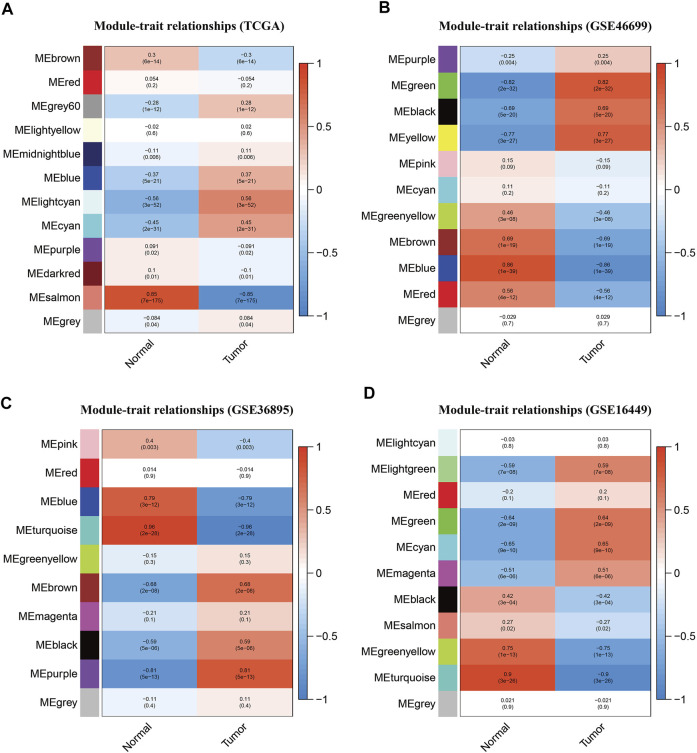
Module-trait relationship heat maps for **(A)** TCGA-KIRC, **(B)** GSE46699, **(C)** GSE36895, and **(D)** GSE16449. Each module displays the association coefficients and corresponding *p*-values between the genes in a specific module and selected clinical features (tumor tissue or normal tissue).

The TCGA-KIRC, GSE46699, GSE36895, and GSE16449 datasets were used to construct a gene co-expression network, and 12, 11, 10, and 11 modules, respectively, were identified in these datasets (Supplementary Figure S1A–D). The module-trait relationship heat map (Figures 1A–D) shows the relationship between each module and the clinical characteristics. The results showed that the salmon module in TCGA-KIRC (*R* = 0.85, *P* = 7e-175), the blue module in GSE46699 (*R* = 0.86, P = 1e-39), the turquoise module in GSE36895 (*R* = 0.96, *P* = 2e-28), and the turquoise module in GSE16449 (*R* = 0.9, *P* = 3e-26) had the strongest association with normal tissues.

### Intersecting Genes Between DEGs and Co-expression Modules

**FIGURE 2:**
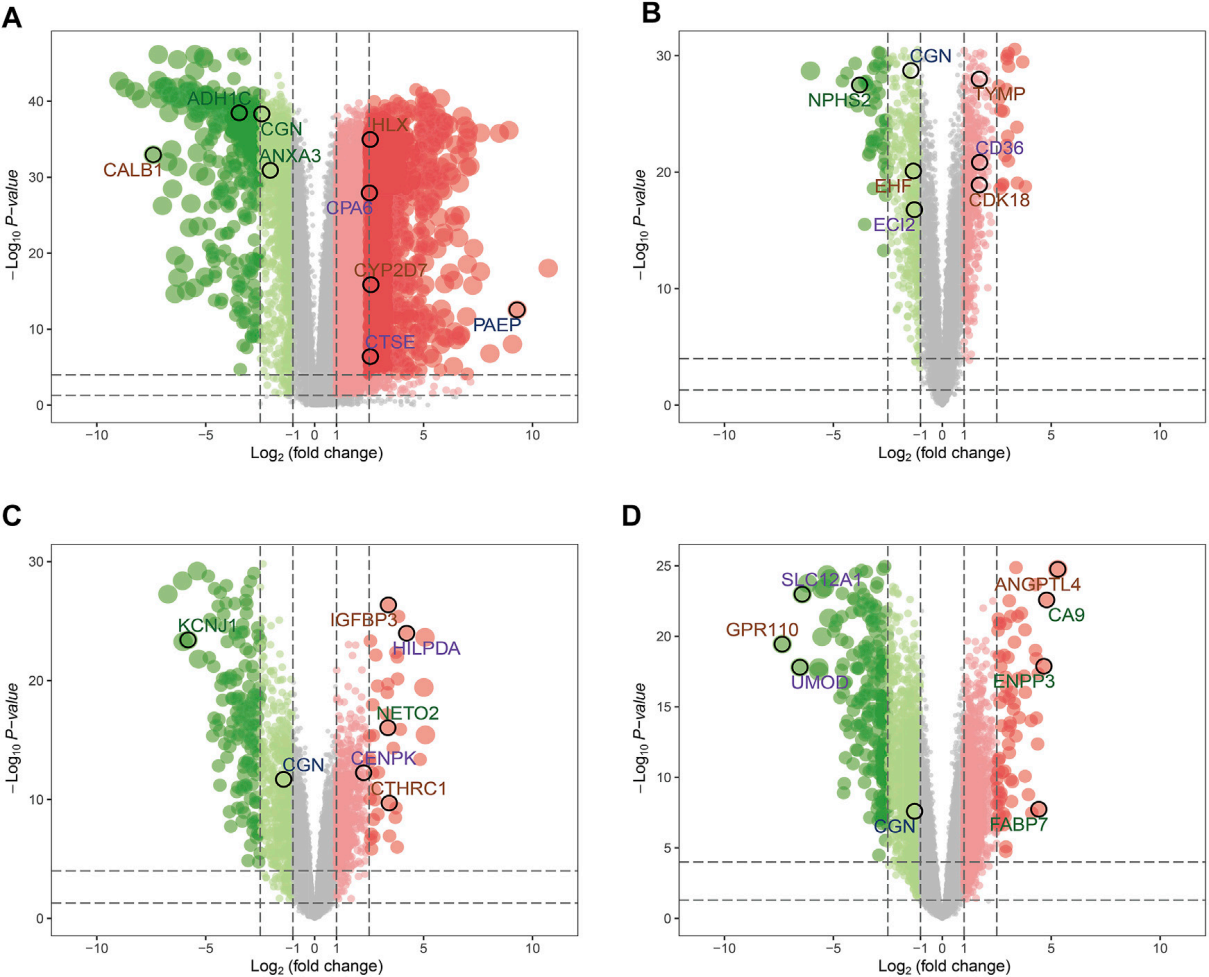
Volcano maps for the differentially expressed genes (DEGs) of **(A)** TCGA-KIRC, **(B)** GSE46699, **(C)** GSE36895, and **(D)** GSE16449.

**FIGURE 3:**
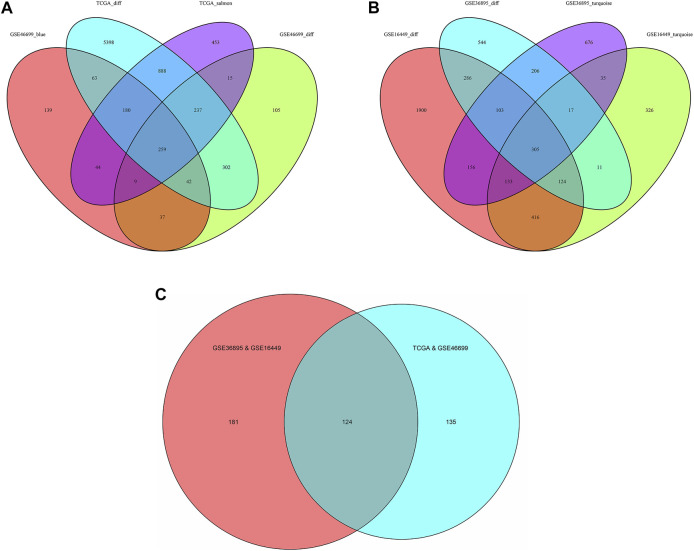
Venn diagram of the genes among the DEG lists and the co-expression modules. **(A)** Among TCGA-KIRC and GSE46699, the results were recorded as TCGA&GSE46699 **(B)** Among GSE16449 and GSE36895, the results were recorded as GSE36895&GSE16449 and **(C)** The intersection of Figure 3. (Abbreviations: TCGA_salmon, GSE46699_blue, GSE16449_ turquoise and GSE36895_turquoise: In WGCNA analysis, the modules with the highest association in the TCGA, GSE46699, GSE16449 and GSE36895 data sets, respectively; TCGA_diff, GSE46699_diff, GSE16449_diff and GSE36895_diff: Differentially expressed genes in the TCGA, GSE46699, GSE16449 and GSE36895 data sets, respectively).

Considering |log_2_FC| ≥1.0 and FDR <0.05 as the threshold values, in the TCGA-KIRC dataset, there were 1902 down-regulated genes and 5467 up-regulated genes; in the GSE46699 dataset, there were 522 down-regulated genes and 484 up-regulated genes; in the GSE36895 dataset, there were 868 down-regulated genes and 728 up-regulated genes; in the GSE16449 dataset, there were 1777 down-regulated genes and 1646 up-regulated genes (Figures 2A–D). Subsequently, the intersection of genes was taken from the modules with the highest association obtained from the WGCNA and DEGs analysis. As shown in Figures 3A–C, there were 124 overlapping genes between the differential genes and the co-expression modules.

### GO, Pathway, and Survival Analysis

To determine the biological functions of the intersecting genes, we performed a GO functional enrichment analysis (Supplementary Figure S2) and a pathway analysis (Supplementary Datasheet S1) on the intersecting genes. In the univariate Cox survival analysis, four overlapping genes were observed to be significantly correlated with the OS (FDR <0.05) (Supplementary Table S1). Multivariate Cox survival analysis also confirmed that the expression of the four genes *CGN, FECH, UCHL1*, and *WT1* were independently correlated with the OS of ccRCC patients.

**FIGURE 4:**
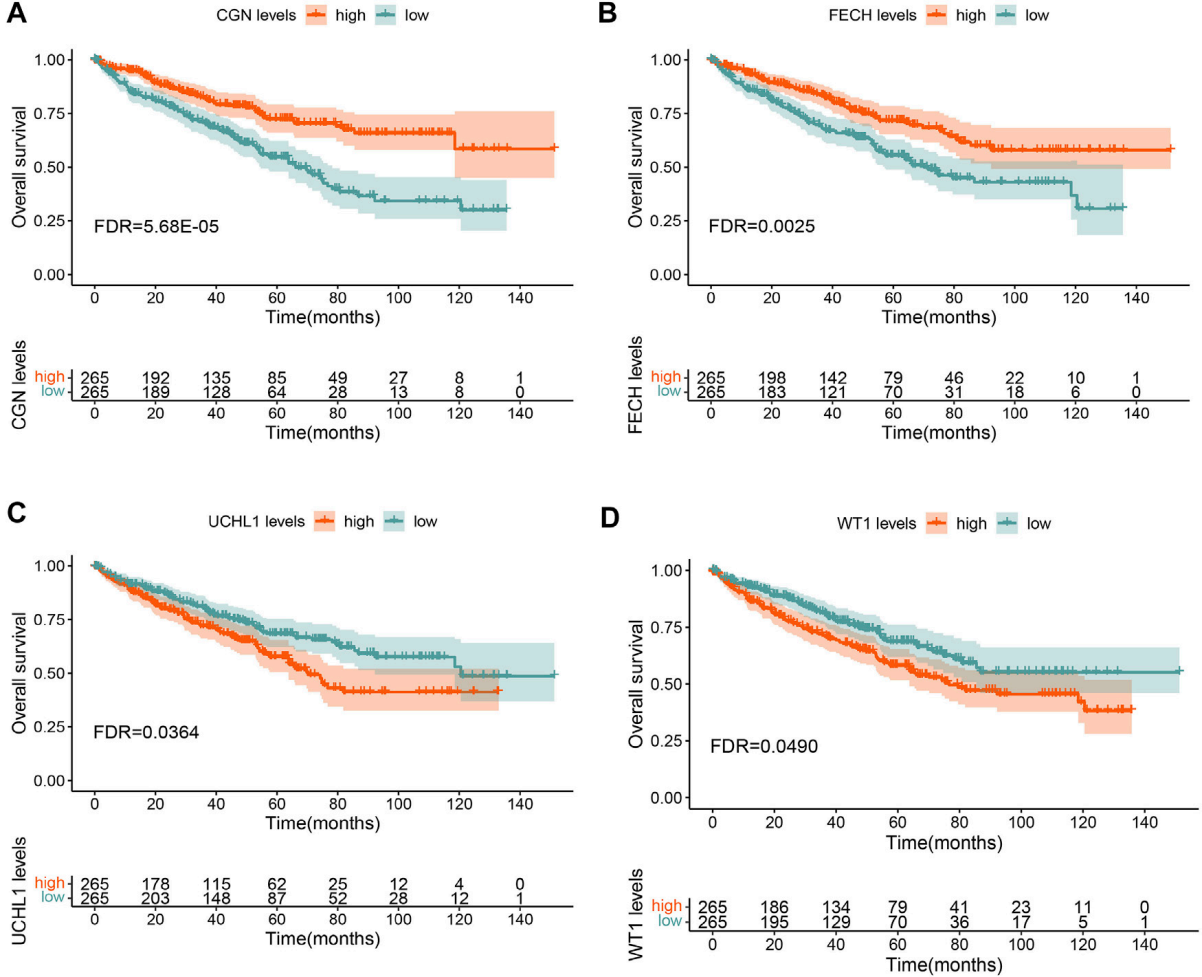
Overall survival (OS) analysis for **(A)** *CGN*, **(B)** FECH, **(C)** UCHL1, and **(D)** WT1 in TCGA-KIRC.

In the KM survival analysis, gene expression was divided into two groups, high and low, according to the median value. The results showed that low expression of *CGN* had the strongest correlation with a poor prognosis and low OS in ccRCC patients. (Supplementary Table S2 and Figure 4). The integrated datasets of TCGA, GSE3, GSE22541 and GSE29609 also showed a significant correlation between *CGN* and prognosis (Supplementary Figure S3). Therefore, the *CGN* gene was selected for further analysis and verification.

### Association and Molecular Characteristics of *CGN* With Clinical Characters

**FIGURE 5:**
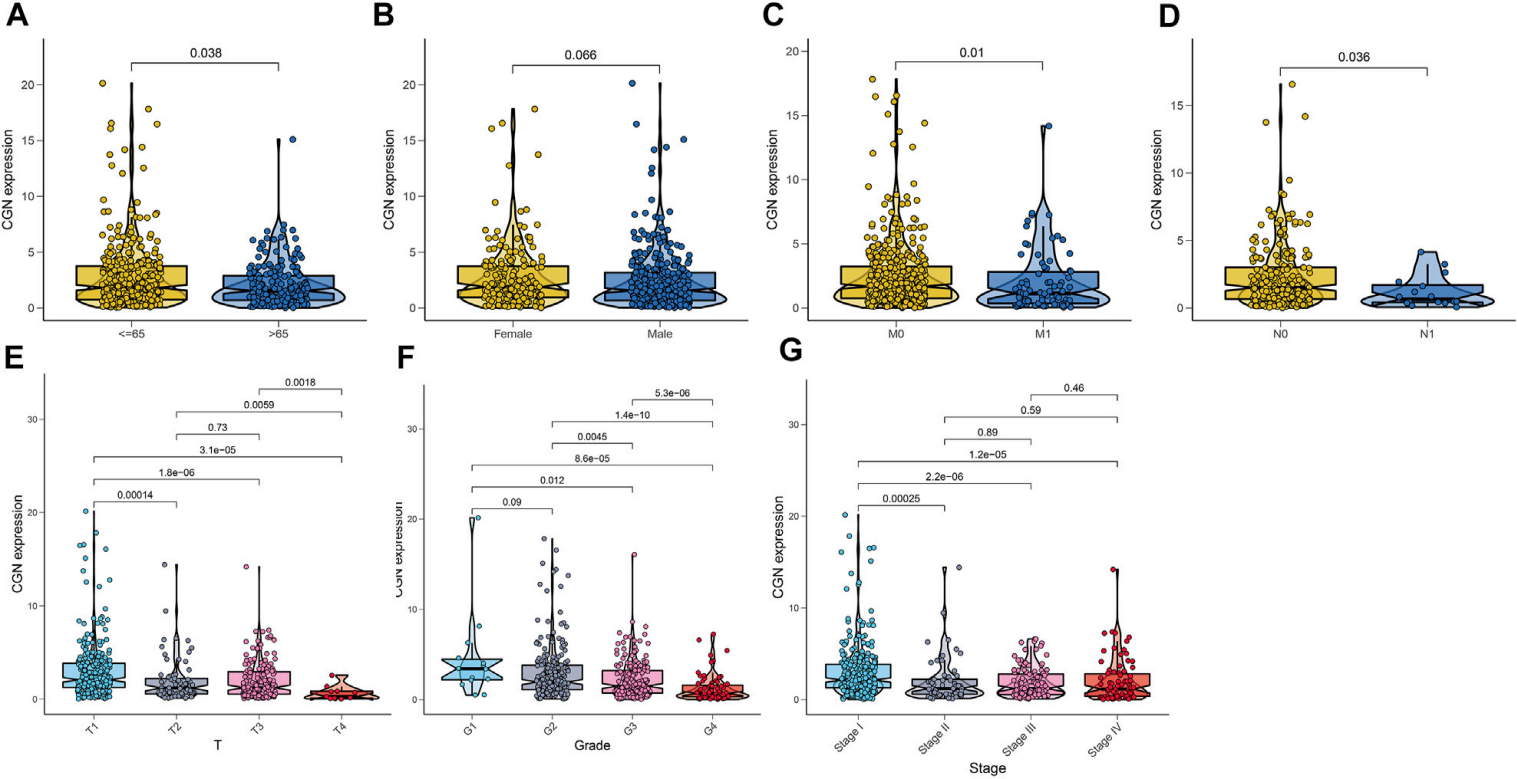
Relationship between *CGN* and **(A)** age, **(B)** gender, **(C)** stage M, **(D)** stage N, **(E)** stage T, **(F)** tumor grade, and **(G)** tumor stage.

We analyzed the relationship between *CGN* expression and age, gender, tumor grade, tumor stage, T stage, N stage, and M stage. The results showed that *CGN* expression decreased significantly with age, advanced tumor stage, high grade, and advanced T, N, and M stages (Figure 5).

### Association Analysis Between *CGN* and TICs

**FIGURE 6:**
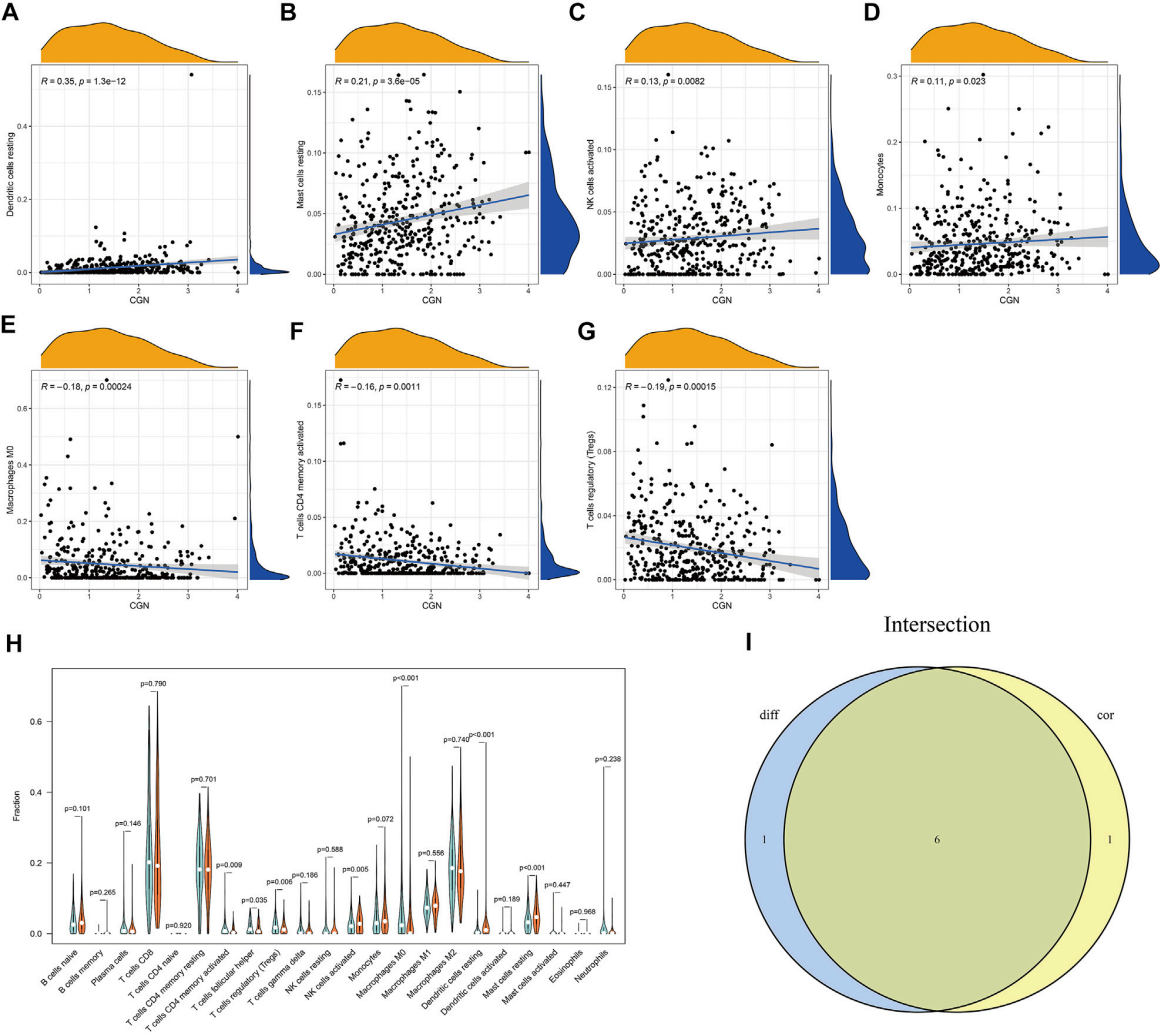
Association analysis between *CGN* and immune cells. **(A–G)** Scatter curve graphs for seven types of immune cells related to *CGN* expression. **(H)** Violin curve graph that shows the differentially expressed immune cells between the two groups with high and low expression of *CGN*. **(I)** Violin graph and scatter graph used to identify six types of immune cells related to *CGN* expression.

The CIBERSORT algorithm was used to analyze the proportion of tumor-infiltrating immune subgroups, and 22 immune cell maps of the ccRCC samples and the association matrix of immune cells were constructed (Supplementary Figure S4). To confirm the association between the expression of *CGN* and immune cells, the Pearson correlation analysis (Figures 6A–G) and the Wilcoxon test (Figure 6H) were performed and the results corresponding to the intersection of these two analyses were used to identify the immune cell types that are most closely related to *CGN*. (Figure 6I). The results showed that six types of TICs are related to the expression of CGN. Among them, activated NK cells, resting dendritic cells, resting monocytes, and resting mast cells were positively correlated with *CGN* expression, while CD4^+^ memory activated T cells, regulatory T cells, and M0 macrophages were negatively correlated with *CGN* expression. These results further support the idea that changes in the expression levels of *CGN* can affect the immune activity of the tumor microenvironment (TME).

### Association Analysis of *CGN* Expression With TMB and PD-L1 Expression

**FIGURE 7:**
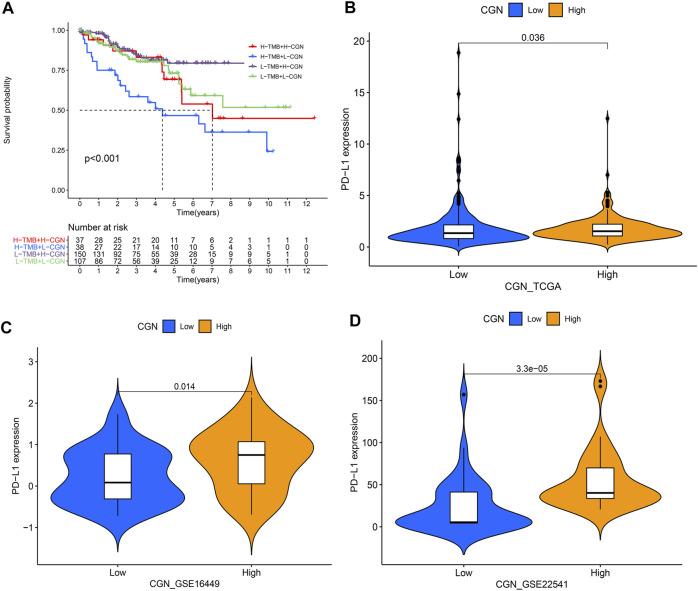
**(A)** Kaplan–Meier curve analyzing the survival of the patient subgroups stratified by CGN and TMB. H, high; L, low. Relationship between *CGN* and PD-L1 expression in **(B)** TCGA, **(C)** GSE16449 and **(D)** GSE22541.

The results showed that the group with a high *CGN* expression had lower TMB (*p* < 0.001; Supplementary Figure S5A). Furthermore, a negative association between *CGN* expression and TMB (*R* = −0.21, *p* < 0.001) was also identified (Supplementary Figure S5B). The KM survival curve results for patients with low TMB showed significant clinical benefits and significantly prolonged rates of survival (*p* < 0.001; Supplementary Figure S5C). After *CGN* and TMB were grouped together, the ccRCC patients with high TMB and low *CGN* had the worst prognosis (*p* < 0.001; Figure 7A). In addition, we divided *CGN* expression into high and low groups according to the median value, and determined the relationship between these two groups and PD-L1 expression. Figures 7B–D shows that the expression of PD-L1 in patients with high *CGN* values is significantly increased (*p* < 0.05).

### Association Analysis Between *CGN* Expression and Existing ccRCC Biomarkers

As Supplementary Figures S6A,B showed, *CGN* expression was positively association with *BAP1* and *SETD2* (*p* < 0.05). In addition, *CGN* expression was negatively correlated with *BIRC5* and *CXCR4* (Supplementary Figures S6C,D).

### Verification of *CGN* Based on the HPA Database and Clinical Samples

**FIGURE 8:**
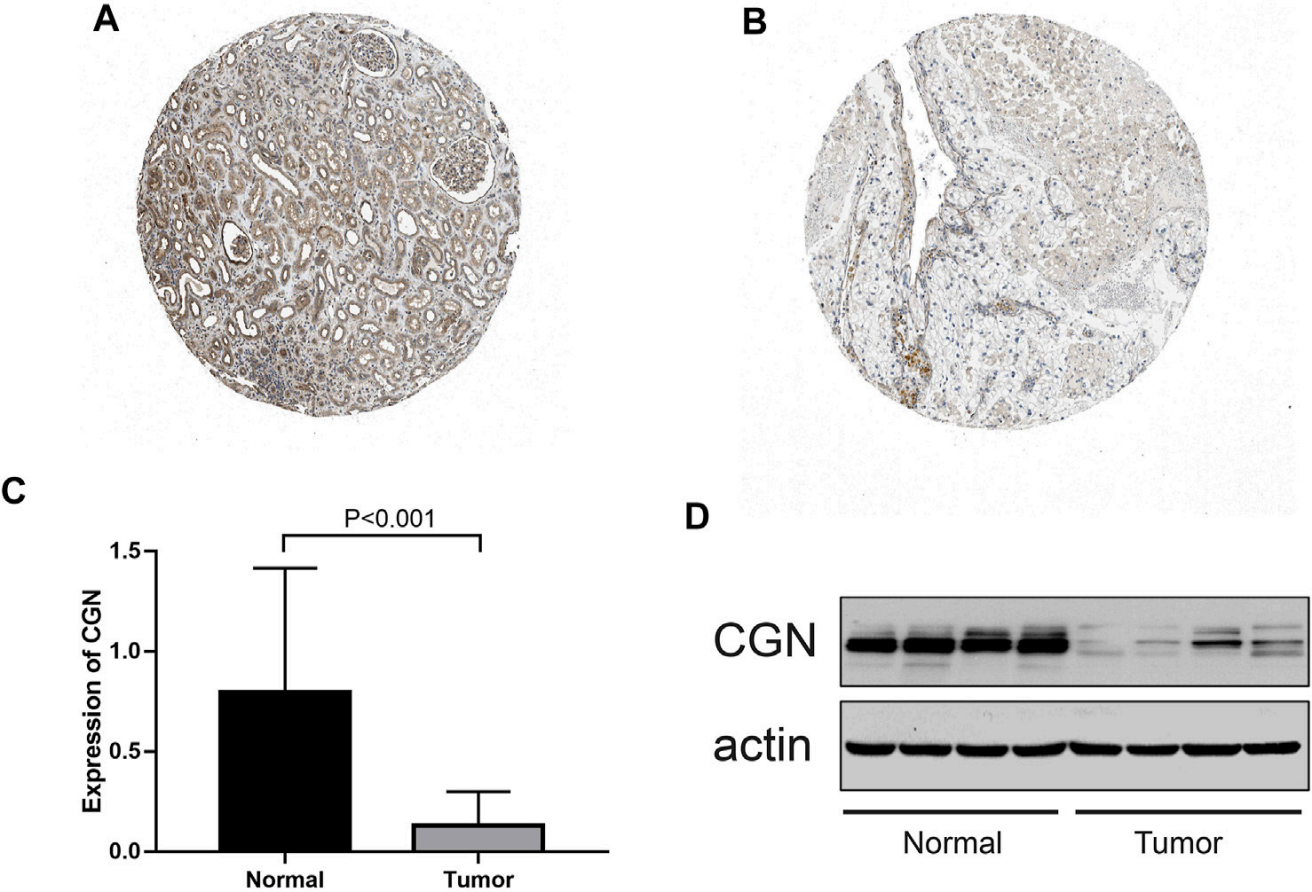
**(A, B)** Immunohistochemistry of the *CGN* gene in normal and tumor tissues from the HPA database. **(C)** RT-PCR analysis of the *CGN* mRNA expression in normal and tumor tissues. **(D)** Expressed *CGN* protein confirmed by western blot analysis in normal and tumor tissues.

According to the HPA database, the *CGN* protein level in the tumor tissues was significantly lower than that in the normal tissues (Figures 8A,B). To better study the expression levels of *CGN* in the normal tissues and tumor tissues of ccRCC patients, 59 samples (Supplementary Tables S3, S4) of each of these tissue types were collected. When compared with the normal tissues, the expression levels of *CGN* in the tumor tissues were significantly reduced (*p* < 0.001; Figure 8C). WB results also showed that protein level of *CGN* in the tumor tissues was significantly lower than that in the normal tissues (*p* < 0.001; Figure 8D).

## Discussion

In this study, a comprehensive bioinformatics analysis identified 124 differentially co-expressed genes in the TCGA-KIRC, GSE46699, GSE36895, and GSE16449 databases. By TCGA and GEO analysis, *CGN* was considered to be the differential gene in ccRCC. Further RT-PCR and WB analyses were performed on *CGN* to verify this. Survival analysis also showed that the expression of *CGN* was mostly correlated with the OS.

*CGN* is a tight junction-related protein that can bind to actin filaments and microtubules and participate in tight junction recombination (Citi et al., 2014). Studies have shown that tight junctions are essential for the barrier functions of the epithelium and the endothelium (Zihni et al., 2016). The downregulation of adhesion functions in tight junctions can lead to an increase in cancer invasion and metastasis (Paschoud et al., 2007), and this has been related to the occurrence of a variety of cancers (Brandner et al., 2015; Zihni et al., 2016). Previous studies have shown that *CGN* is related to the occurrence of lung cancer, and it is highly expressed in lung adenocarcinoma, but significantly reduced in squamous cell carcinoma (Paschoud et al., 2007). Bujko et al. (Bujko et al., 2015) found that when compared with normal tissues, *CGN* is expressed at lower levels in colon adenocarcinoma. In addition, during epithelial-mesenchymal transition in breast cancer model cells, the expression of *CGN* was observed to be downregulated (Papageorgis et al., 2010). Based on these previous findings and our research, it is presumed that the upregulation of *CGN* can inhibit tumor development. Meanwhile, *CGN* is also considered as a prognostic gene of ccRCC in renal clear cell carcinoma (Wu et al., 2021). *BAP1, BIRC5, CXCR4,* and *SETD*2 have been identified as important markers of ccRCC (Petitprez et al., 2021). Our results showed that *CGN* expression was positively associated with tumor suppressors BAP1 (Peña-Llopis et al., 2012) and SETD2 (Li et al., 2016). In addition, CGN expression was negatively associated with the tumor-promoting factors BIRC5 (Liu et al., 2014) and CXCR4 (Wang et al., 2021). These results indicated that CGN was associated with marker genes of existing ccRCC, and also demonstrated the credibility of our results.

The TME is composed of an extracellular matrix and related stromal cells, including immune cells, fibroblasts, and vascular networks. Inflammatory cytokines are already known to predict disease progression (Mihai et al., 2016; Mihai et al., 2019). In addition, the interaction between tumor cells and TME also can help determine tumor progression (Binnewies et al., 2018). Accordingly, we further explored the TICs related to *CGN*. The results showed that *CGN* was positively correlated with resting dendritic cells, resting mast cells, and activated NK cells. Dendritic cells are the most powerful antigen-presenting cells and are the main activating cells of CD4^+^T cells and CD8^+^T cells (Lin et al., 2019). It has been reported that curative tumor regression is mainly mediated by CD8^+^ T cells and cross-presented dendritic cells, suggesting that effective treatment could eliminate tumors through innate and acquired immune responses (Moynihan et al., 2016). Clinical trials for dendritic cell-related tumor immunotherapy have shown promising prospects and achieved success in phase three trials (Kimura et al., 2018). In addition, Guldur et al. (Guldur et al., 2014) found that there are more mast cells in ccRCC tissues than in non-ccRCC tissues. This phenomenon may promote ccRCC angiogenesis and lead to the progression of ccRCC (Chen et al., 2017). Under the guidance of pro-inflammatory chemokines produced by the innate immune and acquired immune cells in the TME, circulating NK cells can be recruited to the site of tumorigenesis (Bernardini et al., 2016). It has been reported that the degree of NK cell infiltration in tumor tissues can predict the prognosis of cancer patients (Mandal et al., 2016). In addition, we also found that *CGN* is negatively correlated with activated CD4^+^ memory T cells, regulatory T cells, and M0 macrophages. CD4^+^memory T cells are important immune cells in the human immune system. They are rapidly activated when antigens meet again and produce a strong response (Strutt et al., 2012). It has been reported that patients who received PD-L1/PD-1 blockade in response to treatment showed a high proportion of CD4^+^ memory T cells before the initiation of treatment (Zuazo et al., 2019). Vahidi et al. (Vahidi et al., 2018) found that an increase in the frequency of CD4^+^ memory cells in the tumor-draining lymph nodes of breast cancer patients could effectively prevent tumor recurrence and play a protective role in tumor progression. Regulatory T cells (Treg) also play a major role in tumor immunity, and the frequency of Treg cells in tumor immune infiltration is often much higher than that in normal tissues. It has been suggested that the common selection of Treg cells by tumors is an important feature of tumor development and a necessary condition for tumor progression in many tumor types (Gallimore et al., 2019). This study found that *CGN* expression is negatively correlated with ccRCC clinical staging and M0 macrophage content, which explains that the latter changes with the progression of tumor staging in patients. M0 macrophages form M1 macrophages under the stimulation of interferon (Billiau and Matthys, 2009), affecting the occurrence and the development of ccRCC. Therefore, the analysis of the proportion of TICs in the ccRCC patients suggests that *CGN* may be involved in the maintenance and regulation of immune activity in the TME.

In recent years, immune checkpoint inhibitors have rewritten the history of tumor treatment and improved drug treatments for ccRCC. TMB and PD-L1 have been reported as new biomarkers for cancer responses to immune checkpoint inhibitors (Rittmeyer et al., 2017; Ready et al., 2019). This study found that *CGN* expression is related to TMB and PD-L1, and the survival analysis curve also shows that the OS of ccRCC patients with a lower expression of *CGN* and elevated TMB is shorter. This also proves that *CGN* could be utilized as a biomarker to improve the precision of ccRCC treatments.

However, this study has some limitations. Firstly, this is a retrospective study, and selection bias cannot be avoided. Secondly, differentially co-expressed genes from RNA-seq data were intersected with differentially co-expressed genes from microarray. The numbers of genes in profiles differed significantly. This should be assessed as a factor that impacts gene intersection. Thirdly, we did not investigate the potential mechanisms underlying the involvement of *CGN* genes in the occurrence and development of ccRCC. Future studies should focus on such mechanisms to improve the efficiency of tumor treatment and the accuracy of diagnosis.

## Conclusion

In summary, through TCGA and GEO screening, and further verification by RT-PCR and WB experiments, it was shown that *CGN* is expressed at low levels in ccRCC and is highly correlated with the immune microenvironment. This study illustrates the clinical role and potential biological characteristics of *CGN* in the treatment of ccRCC.

## Data Availability

The datasets presented in this study can be found in online repositories. The names of the repository/repositories and accession number(s) can be found in the article/Supplementary Material.
