# Supplementary figures and images for: CGN Correlates With the Prognosis and Tumor Immune Microenvironment in Clear Cell Renal Cell Carcinoma
Source: Front Mol Biosci. 2022 Feb 9;9:758974. doi: 10.3389/fmolb.2022.758974 (PMC8865610; doi:10.3389/fmolb.2022.758974)

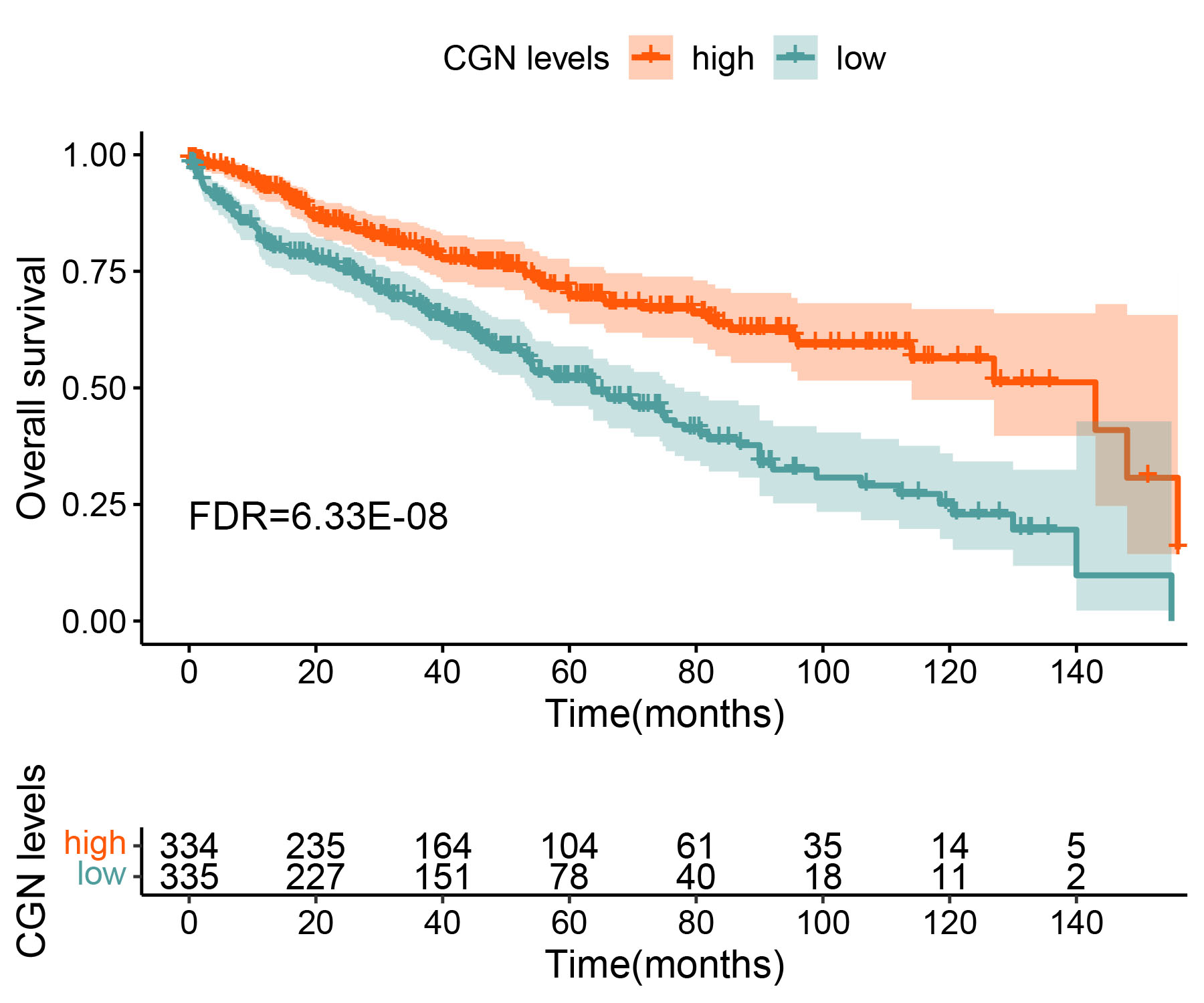

Supplement: Supplementary file 1 [file Image3.JPEG]

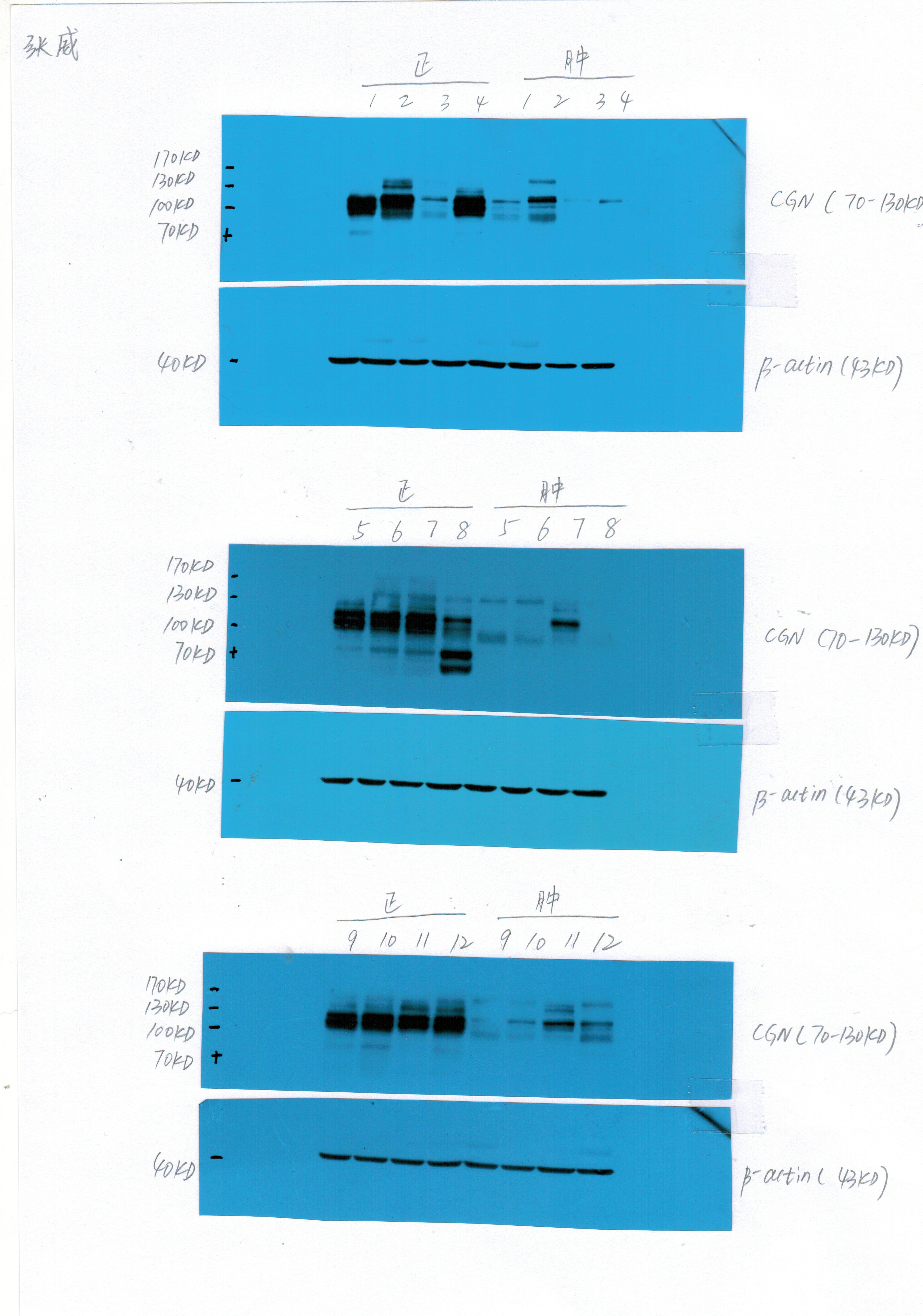

Supplement: Supplementary file 2 [file DataSheet3.ZIP › experiment.WB/CCI20210514_0003.jpg]

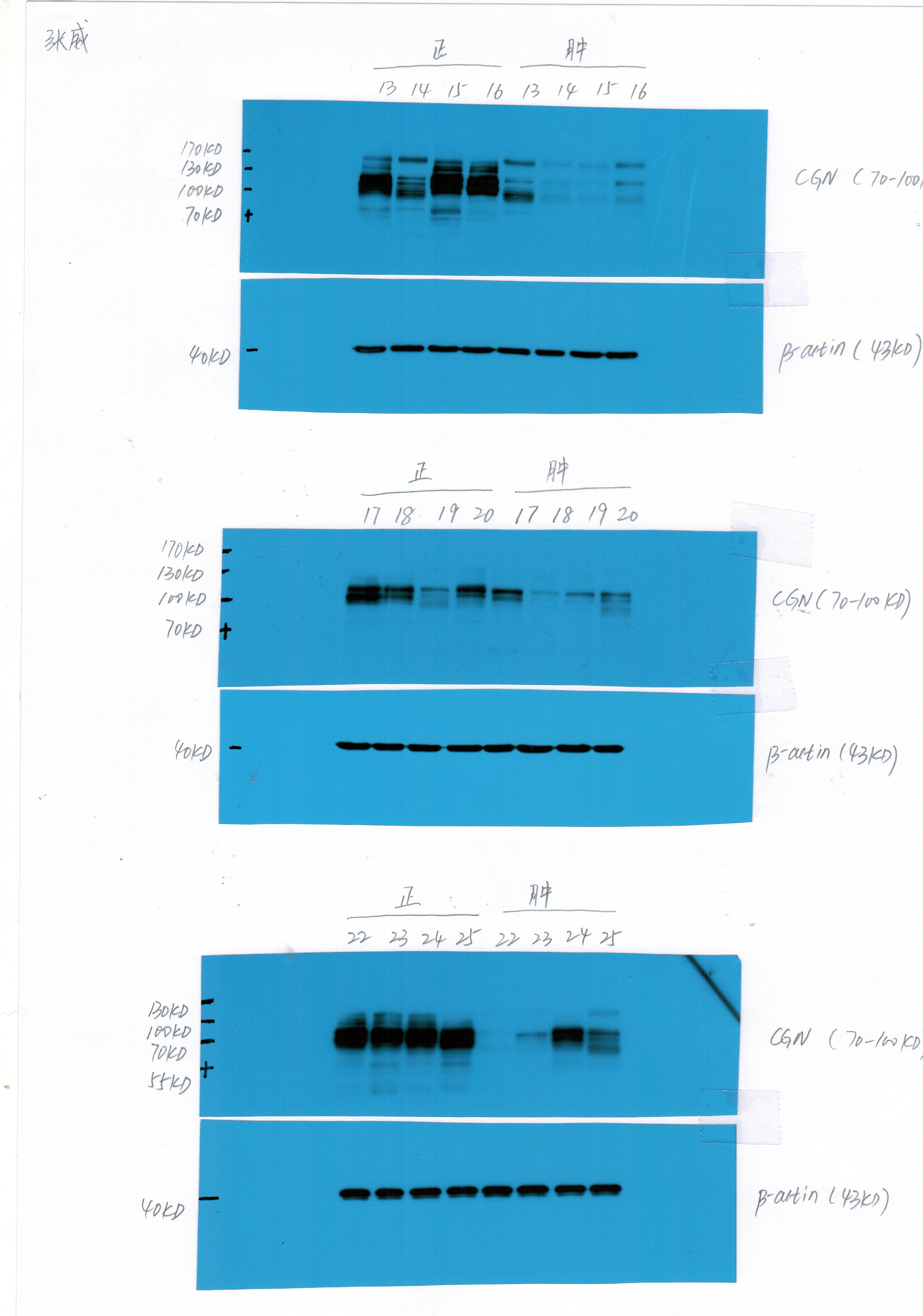

Supplement: Supplementary file 2 [file DataSheet3.ZIP › experiment.WB/CCI20210514_0004.jpg]

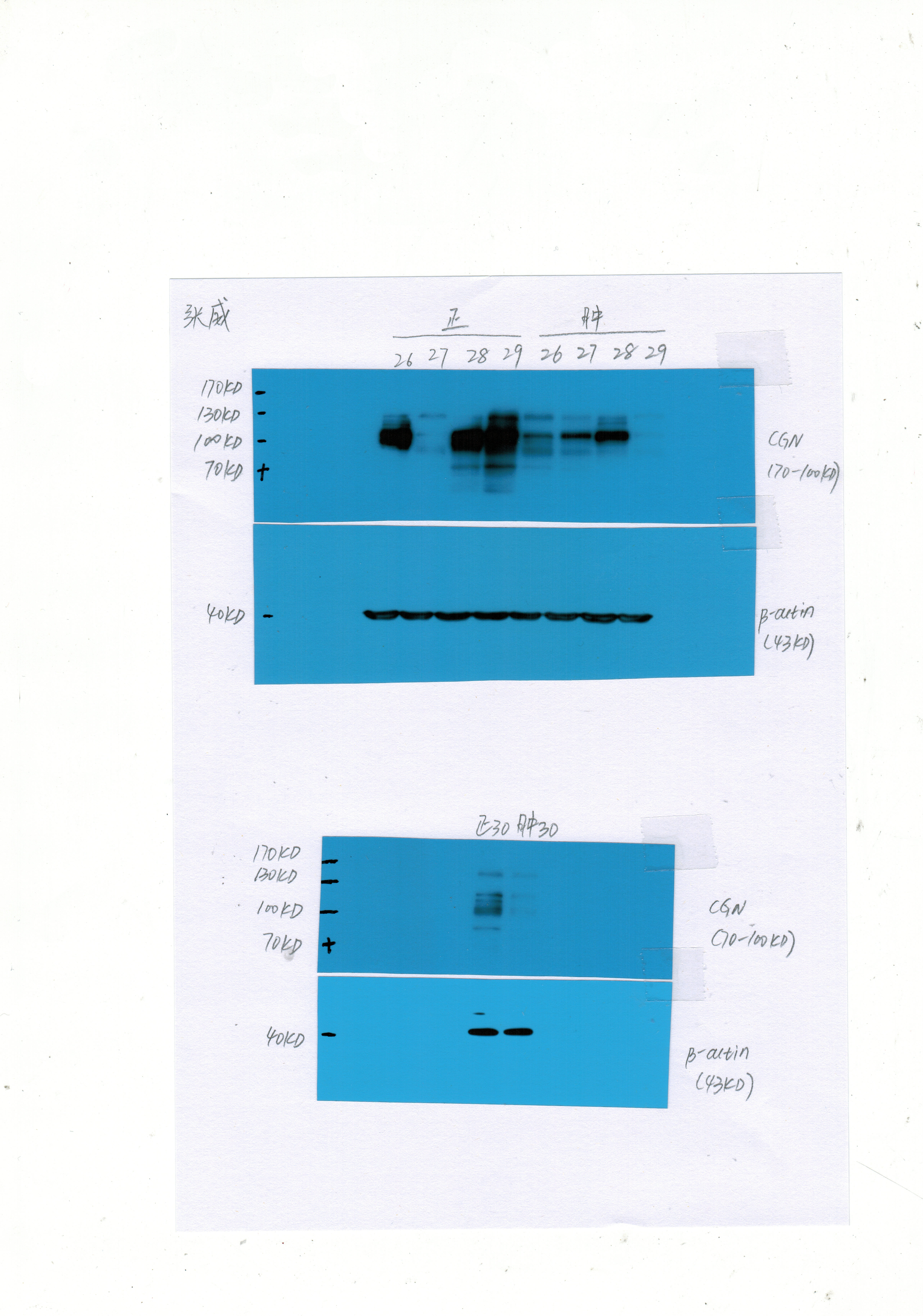

Supplement: Supplementary file 2 [file DataSheet3.ZIP › experiment.WB/CCI20210514_0005.jpg]

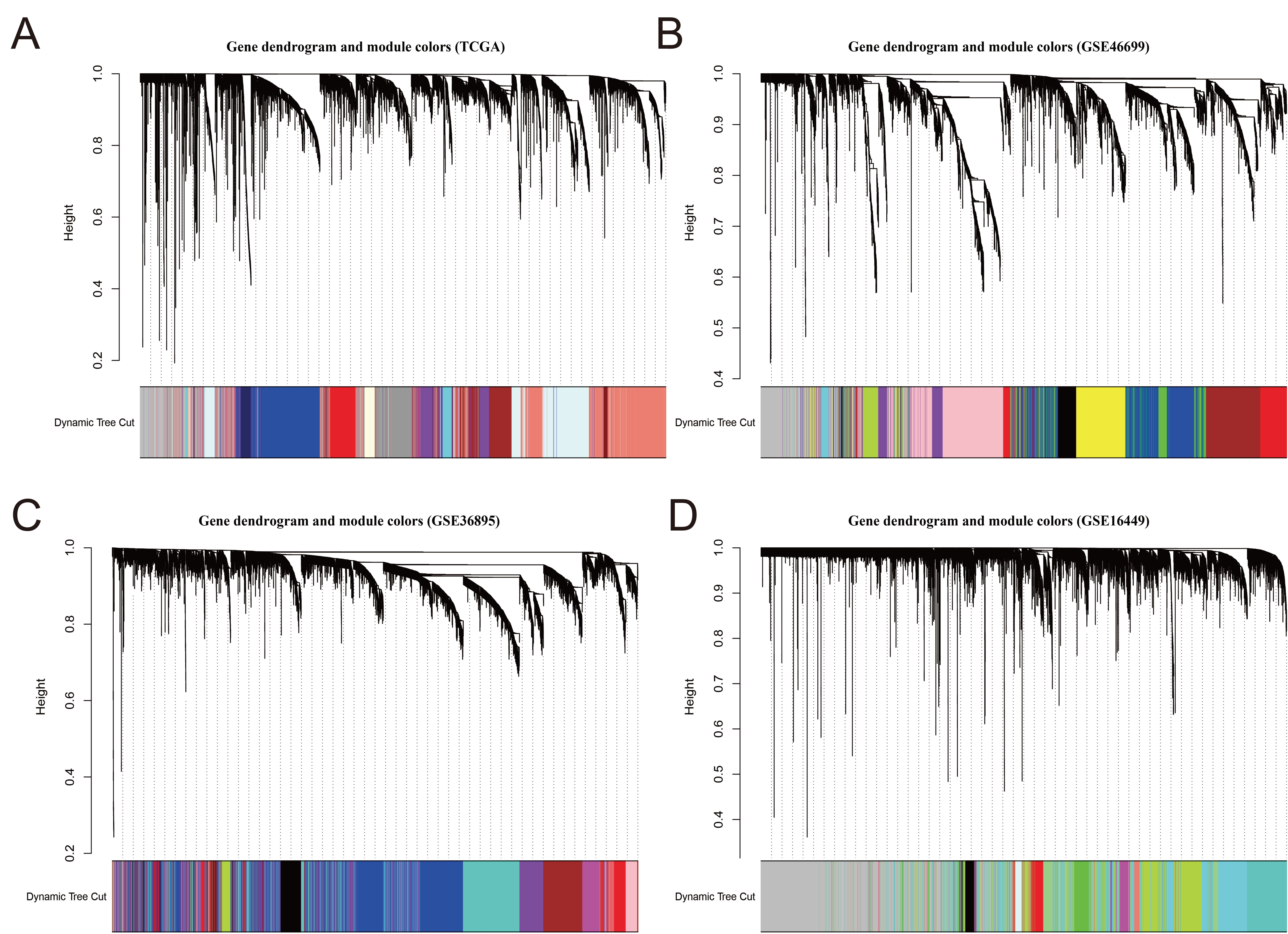

Supplement: Supplementary file 4 [file Image1.JPEG]

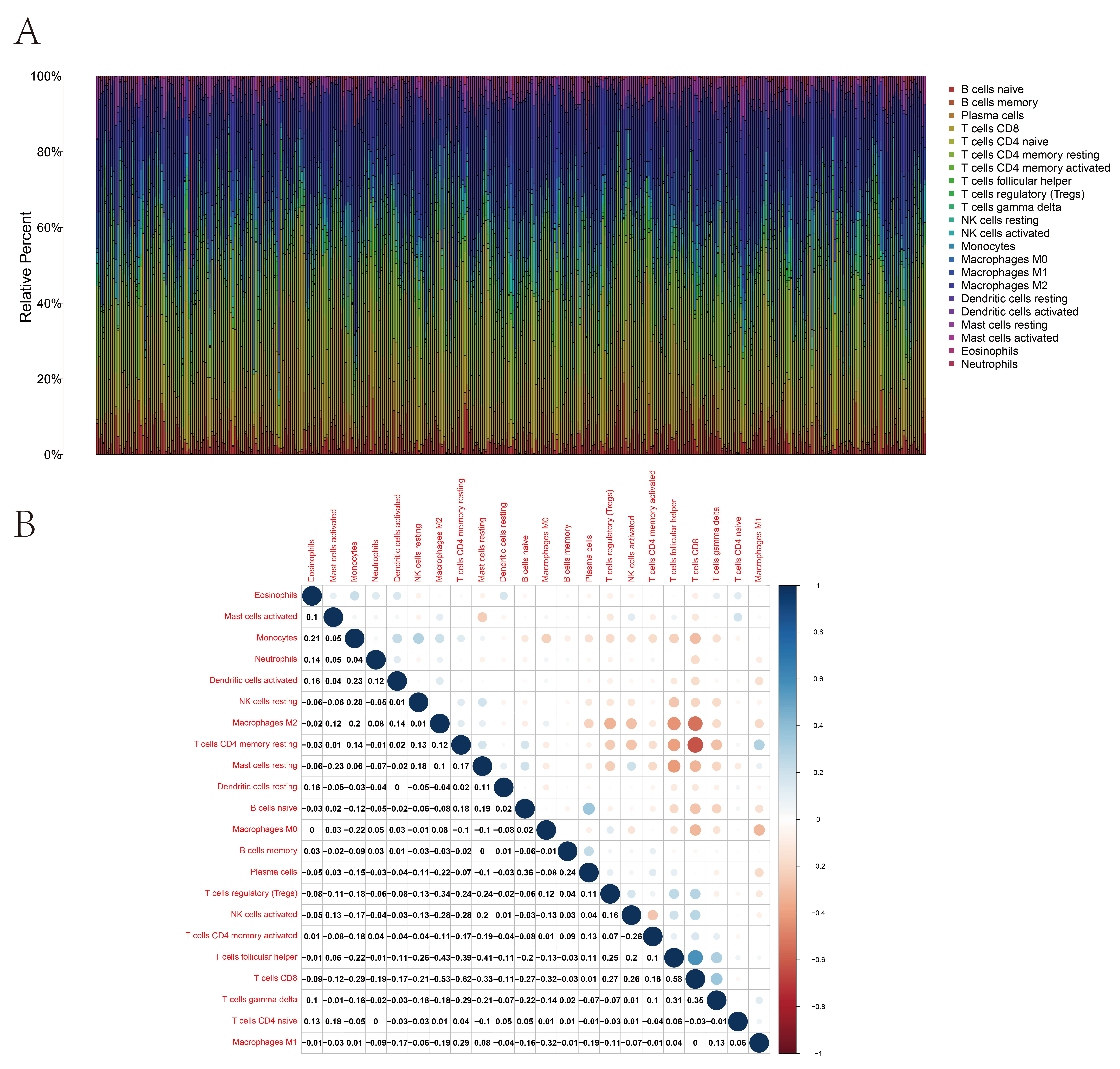

Supplement: Supplementary file 5 [file Image4.JPEG]

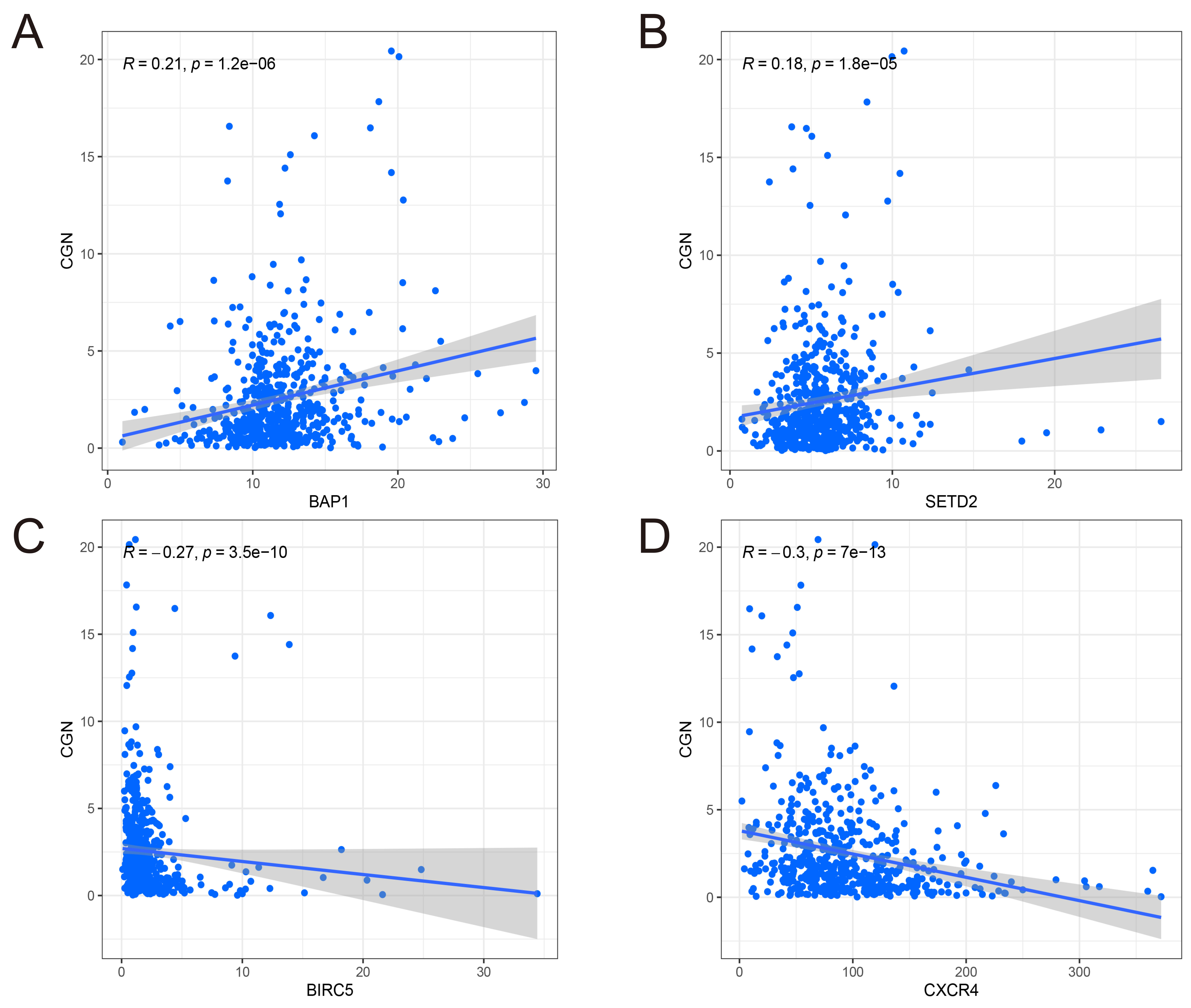

Supplement: Supplementary file 7 [file Image7.JPEG]

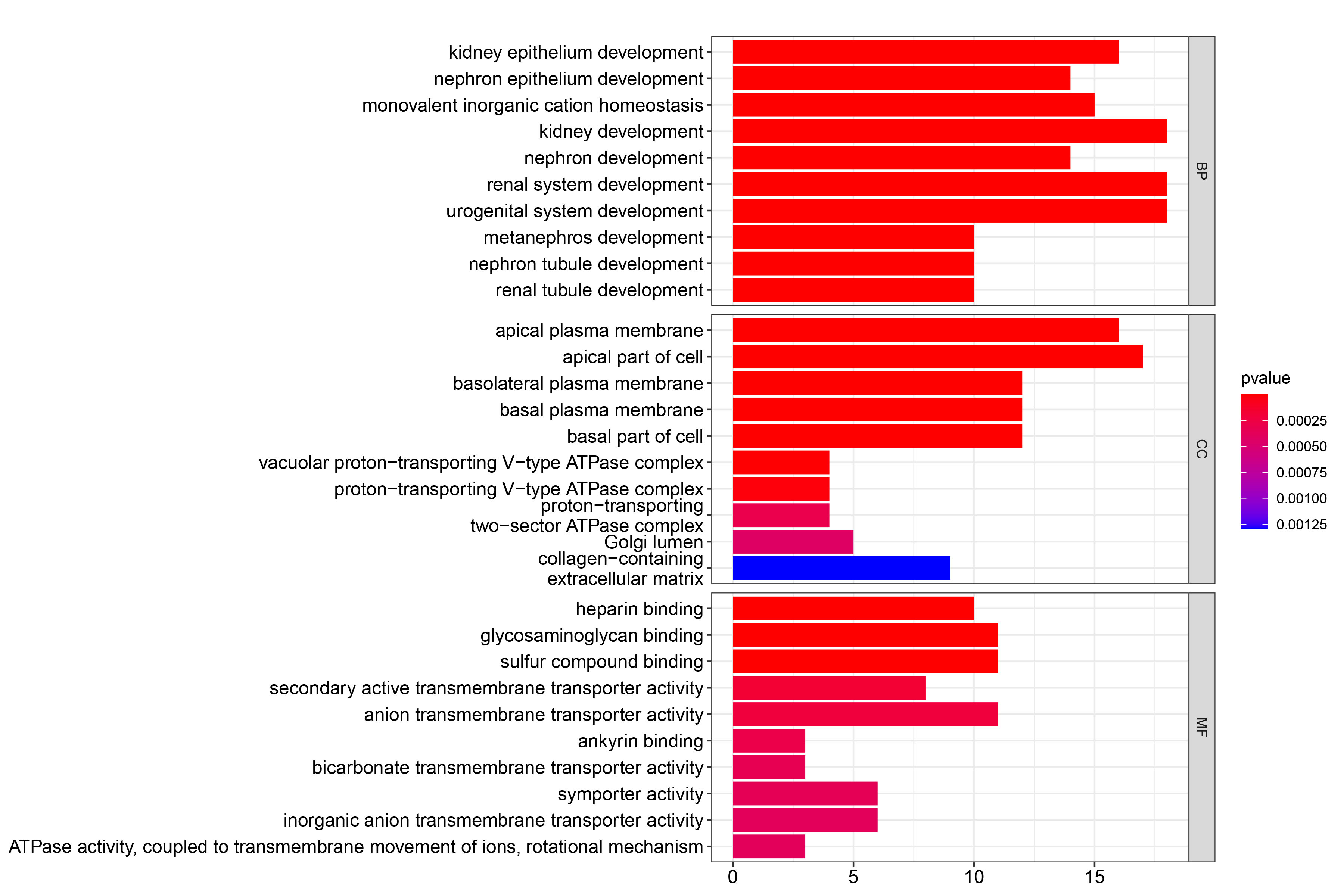

Supplement: Supplementary file 8 [file Image2.JPEG]

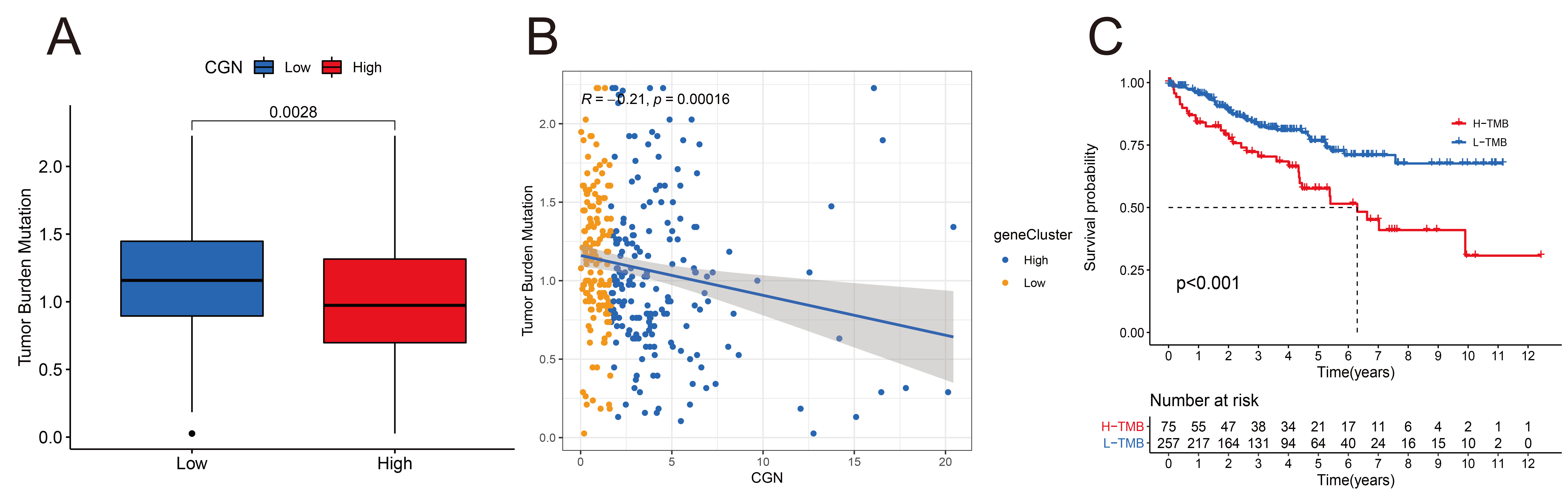

Supplement: Supplementary file 9 [file Image5.JPEG]

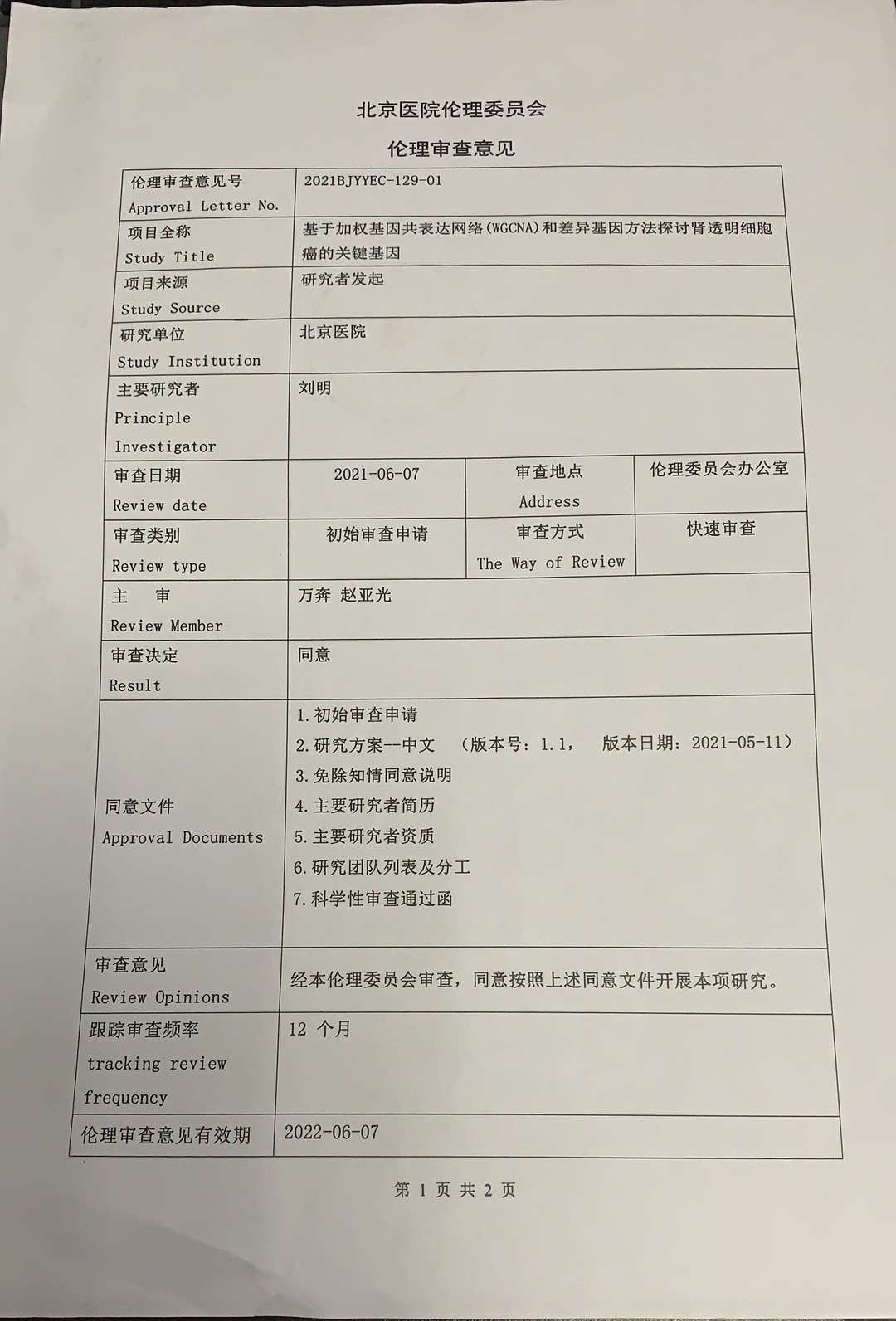

Supplement: Supplementary file 11 [file Image6.JPEG]
