# Supplementary material for: CGN Correlates With the Prognosis and Tumor Immune Microenvironment in Clear Cell Renal Cell Carcinoma
Source: Front Mol Biosci. 2022 Feb 9;9:758974. doi: 10.3389/fmolb.2022.758974 (PMC8865610; doi:10.3389/fmolb.2022.758974)
Supplement: Supplementary file 6 [file DataSheet1.ZIP › Suppl.table/Supplement. Table1.docx]

Supplement. Table 1 Univariate Cox regression of the intersecting genes for overall survival

| Genes | HR | HR.95L | HR.95H | P value | FDR |
| --- | --- | --- | --- | --- | --- |
| ACPP | 0.986491 | 0.679841 | 1.431458 | 0.942917 | 0.966295 |
| ADH1C | 0.995349 | 0.974901 | 1.016226 | 0.659817 | 0.802131 |
| ANXA3 | 0.950287 | 0.898121 | 1.005481 | 0.07669 | 0.31716 |
| AQP2 | 0.991912 | 0.970831 | 1.013451 | 0.458756 | 0.672773 |
| ARNT2 | 0.987099 | 0.95417 | 1.021165 | 0.453204 | 0.672773 |
| ATP6V0A4 | 0.997578 | 0.993787 | 1.001384 | 0.211959 | 0.502406 |
| ATP6V0D2 | 0.997744 | 0.994535 | 1.000962 | 0.16924 | 0.437203 |
| ATP6V1B1 | 0.997642 | 0.992065 | 1.003249 | 0.409027 | 0.656578 |
| ATP6V1C2 | 0.995935 | 0.97621 | 1.016057 | 0.689785 | 0.814603 |
| BMPR1B | 0.984399 | 0.944774 | 1.025686 | 0.453194 | 0.672773 |
| C16orf89 | 0.995468 | 0.972595 | 1.018879 | 0.701719 | 0.815869 |
| C19orf33 | 1.001137 | 0.999843 | 1.002432 | 0.085102 | 0.329771 |
| CALB1 | 0.943896 | 0.820729 | 1.085546 | 0.418304 | 0.656578 |
| CGN | 0.833665 | 0.763942 | 0.909752 | 4.46E-05 | 0.001842 |
| CHGB | 1.008464 | 0.982539 | 1.035072 | 0.525892 | 0.69818 |
| CHL1 | 0.976773 | 0.888336 | 1.074015 | 0.627438 | 0.784653 |
| CLCNKB | 0.997072 | 0.992253 | 1.001915 | 0.235573 | 0.503638 |
| CMTM4 | 0.959209 | 0.926764 | 0.992789 | 0.017682 | 0.156614 |
| COBLL1 | 0.947016 | 0.89115 | 1.006384 | 0.07929 | 0.31716 |
| CRHBP | 0.790743 | 0.652386 | 0.958442 | 0.016734 | 0.156614 |
| DDB2 | 0.993285 | 0.956959 | 1.03099 | 0.723002 | 0.822497 |
| DDN | 0.947591 | 0.669141 | 1.341913 | 0.761698 | 0.843309 |
| DEGS1 | 1.003513 | 0.999831 | 1.007209 | 0.061485 | 0.31716 |
| DIRAS3 | 1.01264 | 0.955114 | 1.073631 | 0.673807 | 0.803416 |
| DMRT2 | 0.994083 | 0.983221 | 1.005065 | 0.289744 | 0.579488 |
| DNER | 1.009099 | 0.997266 | 1.021072 | 0.132311 | 0.415757 |
| DSP | 0.971857 | 0.953598 | 0.990465 | 0.003177 | 0.056819 |
| DUSP9 | 1.038309 | 0.928013 | 1.161713 | 0.51176 | 0.689764 |
| EGF | 0.979779 | 0.932864 | 1.029052 | 0.414491 | 0.656578 |
| EMX1 | 0.950102 | 0.857512 | 1.05269 | 0.327868 | 0.615994 |
| ERBB4 | 1.009187 | 0.876986 | 1.161316 | 0.898423 | 0.952175 |
| ESRP1 | 0.91846 | 0.83661 | 1.008318 | 0.074096 | 0.31716 |
| FAM169A | 0.983955 | 0.817532 | 1.184257 | 0.864149 | 0.931778 |
| FAM171A1 | 0.968602 | 0.932511 | 1.006088 | 0.099628 | 0.363347 |
| FAM3B | 0.728781 | 0.516669 | 1.027971 | 0.071424 | 0.31716 |
| FECH | 0.843848 | 0.7875 | 0.904229 | 1.47E-06 | 0.000157 |
| FGF1 | 0.853082 | 0.739987 | 0.983462 | 0.02854 | 0.18626 |
| FGF9 | 0.991399 | 0.962911 | 1.02073 | 0.561456 | 0.717738 |
| FOXC1 | 0.991123 | 0.946563 | 1.037781 | 0.704016 | 0.815869 |
| FOXI1 | 0.997892 | 0.993198 | 1.002608 | 0.380383 | 0.656578 |
| FXYD4 | 0.968608 | 0.920579 | 1.019143 | 0.219001 | 0.502406 |
| GAS2L3 | 0.98598 | 0.954717 | 1.018266 | 0.3904 | 0.656578 |
| GATA3 | 0.995645 | 0.978994 | 1.012579 | 0.61198 | 0.774342 |
| GLDC | 0.961957 | 0.91753 | 1.008536 | 0.107914 | 0.36598 |
| GMPR | 0.981462 | 0.949699 | 1.014287 | 0.264938 | 0.556819 |
| GPC3 | 0.999198 | 0.98346 | 1.015188 | 0.921113 | 0.955746 |
| GPC5 | 1.133274 | 0.409746 | 3.134401 | 0.809528 | 0.880539 |
| GRB14 | 0.992607 | 0.972201 | 1.013442 | 0.483862 | 0.672773 |
| GSTM3 | 0.958184 | 0.922743 | 0.994986 | 0.026324 | 0.181346 |
| HEPACAM2 | 0.99245 | 0.980968 | 1.004067 | 0.201799 | 0.498639 |
| HIGD1A | 0.99219 | 0.983577 | 1.000879 | 0.077996 | 0.31716 |
| HPGD | 0.920905 | 0.858525 | 0.987819 | 0.021311 | 0.170018 |
| HRG | 0.996829 | 0.977229 | 1.016822 | 0.753899 | 0.842193 |
| HS6ST2 | 1.263534 | 1.076902 | 1.482512 | 0.004124 | 0.056819 |
| HSPA2 | 1.002027 | 0.960709 | 1.045121 | 0.924915 | 0.955746 |
| IGFBP2 | 1.007253 | 0.999756 | 1.014806 | 0.057966 | 0.31716 |
| IRX1 | 1.03277 | 1.000547 | 1.06603 | 0.046176 | 0.286291 |
| IRX2 | 1.112755 | 0.943265 | 1.312701 | 0.205085 | 0.498639 |
| KCNJ1 | 0.942988 | 0.884069 | 1.005833 | 0.074542 | 0.31716 |
| KCNJ10 | 1.077251 | 0.904862 | 1.282482 | 0.402961 | 0.656578 |
| KLHL13 | 0.838212 | 0.719451 | 0.976577 | 0.023573 | 0.171945 |
| KNG1 | 0.986846 | 0.959479 | 1.014994 | 0.356119 | 0.646412 |
| LAD1 | 0.996122 | 0.973046 | 1.019745 | 0.745222 | 0.840068 |
| MAL | 0.997348 | 0.994112 | 1.000594 | 0.109204 | 0.36598 |
| MAN1C1 | 1.002736 | 0.950751 | 1.057564 | 0.919874 | 0.955746 |
| MPPED2 | 0.541623 | 0.342729 | 0.85594 | 0.008635 | 0.107071 |
| MUC1 | 0.998675 | 0.993066 | 1.004316 | 0.644586 | 0.791373 |
| MUC15 | 0.986862 | 0.813065 | 1.19781 | 0.893558 | 0.952175 |
| NAP1L2 | 0.999705 | 0.920541 | 1.085676 | 0.994406 | 0.994406 |
| NDNF | 1.005907 | 0.99764 | 1.014242 | 0.161859 | 0.427031 |
| NELL1 | 1.173398 | 1.023401 | 1.34538 | 0.021938 | 0.170018 |
| NINL | 1.077782 | 0.993697 | 1.168983 | 0.070699 | 0.31716 |
| NLGN1 | 0.964071 | 0.901938 | 1.030485 | 0.281709 | 0.572655 |
| NNMT | 1.000982 | 1.000339 | 1.001626 | 0.00276 | 0.056819 |
| NPHS1 | 0.895734 | 0.70607 | 1.136345 | 0.364375 | 0.646412 |
| NPHS2 | 0.968438 | 0.90353 | 1.038009 | 0.36491 | 0.646412 |
| NR0B2 | 1.000266 | 0.986312 | 1.014417 | 0.970425 | 0.986334 |
| PDE1A | 0.977574 | 0.942985 | 1.013432 | 0.217189 | 0.502406 |
| PRR15 | 0.905999 | 0.803769 | 1.021232 | 0.106089 | 0.36598 |
| PTPRO | 1.061487 | 1.013355 | 1.111905 | 0.011725 | 0.121158 |
| RAB25 | 0.965184 | 0.931114 | 1.000501 | 0.05328 | 0.314607 |
| RALYL | 1.201366 | 0.867294 | 1.66412 | 0.269793 | 0.557572 |
| RHBG | 0.990128 | 0.967587 | 1.013194 | 0.398477 | 0.656578 |
| RHCG | 0.99939 | 0.997754 | 1.001029 | 0.465668 | 0.672773 |
| RNF150 | 0.973354 | 0.858324 | 1.103799 | 0.673832 | 0.803416 |
| S100A2 | 0.999882 | 0.987714 | 1.012199 | 0.984866 | 0.992873 |
| SCNN1A | 0.993686 | 0.984668 | 1.002786 | 0.173245 | 0.438415 |
| SCNN1G | 1.00226 | 0.99593 | 1.008631 | 0.484901 | 0.672773 |
| SERPINA5 | 0.996076 | 0.980139 | 1.012272 | 0.632785 | 0.784653 |
| SFRP1 | 0.986914 | 0.96197 | 1.012504 | 0.313188 | 0.597465 |
| SHISA3 | 0.996337 | 0.986829 | 1.005936 | 0.453139 | 0.672773 |
| SLC12A1 | 0.97455 | 0.916923 | 1.035798 | 0.407122 | 0.656578 |
| SLC12A3 | 0.993552 | 0.981475 | 1.005776 | 0.29981 | 0.586447 |
| SLC16A1 | 1.018234 | 1.005745 | 1.030879 | 0.004109 | 0.056819 |
| SLC16A5 | 0.981998 | 0.93164 | 1.035079 | 0.498836 | 0.679733 |
| SLC16A7 | 0.984967 | 0.954412 | 1.016499 | 0.346125 | 0.64059 |
| SLC19A2 | 0.978114 | 0.943076 | 1.014454 | 0.234469 | 0.503638 |
| SLC26A4 | 0.870746 | 0.5887 | 1.287922 | 0.488303 | 0.672773 |
| SLC4A1 | 0.99361 | 0.98342 | 1.003904 | 0.222842 | 0.502406 |
| SLC4A9 | 0.979366 | 0.923983 | 1.038068 | 0.482677 | 0.672773 |
| SLFN13 | 0.989488 | 0.935458 | 1.046639 | 0.712243 | 0.81776 |
| SLIT2 | 0.990024 | 0.957569 | 1.02358 | 0.5555 | 0.717521 |
| SOST | 0.755135 | 0.39033 | 1.460887 | 0.404183 | 0.656578 |
| SOSTDC1 | 0.943811 | 0.883113 | 1.008682 | 0.088178 | 0.331336 |
| SPINK1 | 0.997567 | 0.990729 | 1.004453 | 0.487631 | 0.672773 |
| TBC1D24 | 0.960696 | 0.865313 | 1.066592 | 0.452301 | 0.672773 |
| TCEAL2 | 0.95251 | 0.880037 | 1.030952 | 0.228202 | 0.503638 |
| TFAP2B | 0.701677 | 0.433496 | 1.135766 | 0.149344 | 0.427031 |
| TFCP2L1 | 0.987003 | 0.969117 | 1.005219 | 0.160884 | 0.427031 |
| TMC4 | 0.985892 | 0.967733 | 1.004391 | 0.134115 | 0.415757 |
| TMEM140 | 0.988314 | 0.980581 | 0.996107 | 0.003356 | 0.056819 |
| TMEM30B | 0.970692 | 0.93299 | 1.009918 | 0.14111 | 0.426773 |
| TMEM45B | 0.921075 | 0.824646 | 1.028779 | 0.145087 | 0.427031 |
| TMEM61 | 0.958749 | 0.904644 | 1.016091 | 0.155212 | 0.427031 |
| TMPRSS2 | 0.983952 | 0.954142 | 1.014693 | 0.302682 | 0.586447 |
| TNFAIP6 | 0.995372 | 0.990493 | 1.000275 | 0.06425 | 0.31716 |
| TSPAN8 | 1.01369 | 0.996588 | 1.031086 | 0.117281 | 0.382706 |
| TYMS | 0.96576 | 0.940654 | 0.991536 | 0.009528 | 0.107411 |
| TYRP1 | 0.893605 | 0.629429 | 1.268658 | 0.529265 | 0.69818 |
| UCHL1 | 1.013886 | 1.008078 | 1.019726 | 2.54E-06 | 0.000157 |
| UMOD | 0.998642 | 0.994345 | 1.002958 | 0.536841 | 0.700719 |
| VTCN1 | 0.973515 | 0.937995 | 1.01038 | 0.15694 | 0.427031 |
| WNK4 | 1.011734 | 0.92616 | 1.105216 | 0.795849 | 0.873321 |
| WT1 | 1.107216 | 1.052654 | 1.164607 | 7.81E-05 | 0.002421 |

Abbreviations: HR, hazard ratio; 95%CI, 95% confidence intervals.
