# Supplementary material for: CGN Correlates With the Prognosis and Tumor Immune Microenvironment in Clear Cell Renal Cell Carcinoma
Source: Front Mol Biosci. 2022 Feb 9;9:758974. doi: 10.3389/fmolb.2022.758974 (PMC8865610; doi:10.3389/fmolb.2022.758974)
Supplement: Supplementary file 6 [file DataSheet1.ZIP › Suppl.table/Supplement. Table2.docx]

Supplement. Table2 Multivariate Cox regression and K-M survival analysis of the prognostic genes for overall survival

| Genes |  | Multivariate analysis |  |  | K-M survival analysis | |
| --- | --- | --- | --- | --- | --- | --- |
|  |  | HR (95%CI) | P value |  | P value | FDR |
| CGN |  | 0.885 (0.814-0.962) | <0.05 |  | 1.83E-06 | 5.68E-05 |
| FECH |  | 0.894 (0.832-0.961) | <0.05 |  | 0.0002 | 0.0025 |
| UCHL1 |  | 1.010 (1.003-1.016) | <0.05 |  | 0.0065 | 0.0364 |
| WT1 |  | 1.059 (1.004-1.117) | <0.05 |  | 0.0091 | 0.0490 |

Abbreviations: HR, hazard ratio; 95%CI, 95% confidence intervals.
