# Supplementary material for: CGN Correlates With the Prognosis and Tumor Immune Microenvironment in Clear Cell Renal Cell Carcinoma
Source: Front Mol Biosci. 2022 Feb 9;9:758974. doi: 10.3389/fmolb.2022.758974 (PMC8865610; doi:10.3389/fmolb.2022.758974)
Supplement: Supplementary file 6 [file DataSheet1.ZIP › Suppl.table/Supplement. Table3.docx]

Supplement. Table3 Clinicopathological parameters of patients in PCR experiments.

| **Patient id** | **Age** | **Gender** | **Stage T** | **Stage N** | **Stage M** | **Fuhrman** |
| --- | --- | --- | --- | --- | --- | --- |
| 1 | 58 | Male | T1a | 0 | 0 | II |
| 2 | 66 | Female | T1a | 0 | 0 | II |
| 3 | 63 | Male | T1a | 0 | 0 | II |
| 4 | 61 | Female | T1b | 0 | 0 | II |
| 5 | 60 | Male | T3a | 0 | 0 | III |
| 6 | 52 | Male | T1a | 0 | 0 | II |
| 7 | 58 | Male | T1a | 0 | 0 | III |
| 8 | 44 | Male | T1a | 0 | 0 | II |
| 9 | 46 | Male | T1a | 0 | 0 | II |
| 10 | 50 | Male | T3c | 0 | 0 | IV |
| 11 | 76 | Female | T1 | 0 | 0 | III |
| 12 | 38 | Male | T1 | 0 | 0 | II |
| 13 | 52 | Female | T1 | 0 | 0 | II |
| 14 | 65 | Male | T1 | 0 | 0 | III |
| 15 | 71 | Female | T1 | 0 | 0 | II |
| 16 | 66 | Male | T1 | 0 | 0 | II |
| 17 | 66 | Male | T3 | 0 | 0 | III |
| 18 | 66 | Male | T1 | 0 | 0 | II |
| 19 | 65 | Male | T1 | 0 | 0 | II |
| 20 | 50 | Female | T3 | 0 | 0 | III |
| 21 | 62 | Male | T1 | 0 | 0 | II |
| 22 | 62 | Male | T3 | 0 | 0 | III |
| 23 | 47 | Male | T3 | 0 | 0 | II |
| 24 | 84 | Female | T1 | 0 | 0 | II |
| 25 | 73 | Female | T1 | 0 | 0 | II |
| 26 | 54 | Female | T1 | 0 | 0 | I |
| 27 | 56 | Male | T2 | 0 | 0 | III |
| 28 | 66 | Male | T3 | 0 | 0 | III |
| 29 | 46 | Male | T1 | 0 | 0 | II |
| 30 | 59 | Male | T1 | 0 | 0 | II |
